# Watershed Urbanization Alters the Composition and Function of Stream Bacterial Communities

**DOI:** 10.1371/journal.pone.0022972

**Published:** 2011-08-12

**Authors:** Si-Yi Wang, Elizabeth B. Sudduth, Matthew D. Wallenstein, Justin P. Wright, Emily S. Bernhardt

**Affiliations:** 1 Biology Department, Duke University, Durham, North Carolina, United States of America; 2 Natural Resource Ecology Lab, Colorado State University, Fort Collins, Colorado, United States of America; Argonne National Laboratory, United States of America

## Abstract

Watershed urbanization leads to dramatic changes in draining streams, with urban streams receiving a high frequency of scouring flows, together with the nutrient, contaminant, and thermal pollution associated with urbanization. These changes are known to cause significant losses of sensitive insect and fish species from urban streams, yet little is known about how these changes affect the composition and function of stream microbial communities. Over the course of two years, we repeatedly sampled sediments from eight central North Carolina streams affected to varying degrees by watershed urbanization. For each stream and sampling date, we characterized both overall and denitrifying bacterial communities and measured denitrification potentials. Denitrification is an ecologically important process, mediated by denitrifying bacteria that use nitrate and organic carbon as substrates. Differences in overall and denitrifying bacterial community composition were strongly associated with the gradient in urbanization. Denitrification potentials, which varied widely, were not significantly associated with substrate supply. By incorporating information on the community composition of denitrifying bacteria together with substrate supply in a linear mixed-effects model, we explained 45% of the variation in denitrification potential (*p*-value<0.001). Our results suggest that (1) the composition of stream bacterial communities change in response to watershed urbanization and (2) such changes may have important consequences for critical ecosystem functions such as denitrification.

## Introduction

Streams occupy low lying points in landscapes and are thus strongly affected by the detrimental impacts of watershed urbanization (reviewed by [1]). An extensive body of research has documented significant losses of sensitive insect and fish species from streams in response to urbanization (reviewed by [2]). In contrast, relatively little is known about how urbanization affects bacterial community composition in streams [3]. Given that bacteria mediate many of the biogeochemical transformations underpinning ecosystem functioning [4], changes to the composition of stream bacterial communities in response to urbanization have the potential to alter ecosystem functioning in streams [5].

There are reasons to expect watershed urbanization to alter bacterial community composition in streams – urban streams are highly stressful environments that receive severe and frequent physical disturbance through scouring flows, together with increased stream water temperatures and contaminant (e.g., heavy metals) and nutrient inputs [1], [6]. Physical disturbance, as well as thermal and chemical changes, have been found to strongly regulate bacterial community composition in streams (reviewed by [7]) and terrestrial systems [5], [8], [9]. We therefore expected watershed urbanization to cause substantial changes to the composition of stream bacterial communities. In the single published study on this topic to date, Lear *et al.* found that bacterial community composition in stream biofilms was significantly different between streams in urban versus rural watersheds [10].

It is less clear how urbanization might affect the composition and function of bacterial functional groups (i.e., groups whose members all share the ability to perform a particular function). One particularly important functional group and function in streams is denitrifying bacteria (denitrifiers) and denitrification. Denitrification is the transformation of nitrate to nitrogen gas and is one of the few ways to permanently remove nitrate from surface waters. The global supply of nitrate has more than doubled over the last century [11], particularly in urban streams [1], [12], leading to serious water quality and human health problems [13]–[15]. Denitrification by stream denitrifiers can play a key role in mitigating nitrogen pollution by preventing nitrate from entering downstream ecosystems [12], [16], [17]. Our goal in the study was to examine whether watershed urbanization affects the composition of overall bacterial communities and the composition and function of denitrifier communities in streams.

Most denitrifiers are facultative anaerobes, using oxygen as the electron acceptor for organic matter oxidation whenever available and nitrate as the next best alternative electron acceptor under hypoxic or anoxic conditions. Denitrification is thus expected to be highest in habitats with low dissolved oxygen and high nitrate and organic carbon substrate supply. Urban streams tend to have low oxygen and high substrate concentrations [1], making them seemingly ideal places for denitrification. Urbanization, however, affects more than just oxygen and substrate concentrations; it also imposes many stressors on stream inhabitants, such as contamination, high temperatures, and hydrologic disturbances [1]. These stressors may drive changes in denitrifier community composition [18] that could, in turn, lead to altered denitrification rates [19].

**Figure 1:**
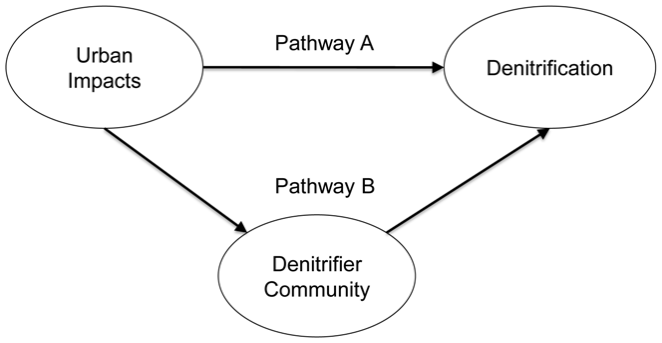
Conceptual diagram describing two causal mechanisms through which watershed urbanization might alter denitrification rates in streams. The first mechanism (pathway A) focuses on the direct effect of urbanization on denitrification through changes to nitrate and organic carbon concentrations. The second mechanism (pathway B) focuses on the indirect effect of urbanization on denitrification through changes to denitrifier community composition.

We propose two mechanisms by which watershed urbanization could affect stream denitrification. The first mechanism focuses on direct effects of urbanization on denitrification through changes to nitrate and organic carbon concentrations. The second mechanism focuses on indirect effects of urbanization on denitrification through changes to denitrifier community composition (Fig. 1). To compare the relative importance of the two mechanisms, we examined both denitrifier community composition and substrate concentrations as potential controls of denitrification potential in streams affected to varying degrees by watershed urbanization.

## Methods

### Study streams

We surveyed microbial communities and measured denitrification potentials in eight study streams located in the Raleigh-Durham area in the Piedmont region of North Carolina (USA) (Table 1). We selected the study streams to represent a gradient of watershed urbanization, with percent impervious cover (% IC) ranging from 1 to 39%. Watershed land cover metrics were calculated based on the 2001 National Land Cover Dataset and the associated Impervious Surface Cover dataset, both from the United States Geological Survey Seamless Server. Within each study stream, we established a permanent study reach from which to collect samples during the course of the research effort (June 2008 to July 2009). Further details on the physical conditions (e.g., total degree-days, hydrologic flashiness, etc.) and the macroinvertebrate communities at each site can be found in [20], [21].

**Table 1:** Study streams, ranked in order of percent impervious cover in the watershed.

Stream	Watershed impervious cover (%)	Watershed develop-ment (%)	Nitrate (mg/L)^1^	TOC (mg/L)^1^	Total metals^2^	Sediment d50^3^	Total degreedays^4^	Flashi-ness^5^	EPT richness^6^
Mud Creek	0.5	4.4	0.111	4.775	16	13	11018	0.04	12.0
Stony	3.4	24.4	0.200	4.173	29	111	10691	0.01	9.0
Lower Mud	9.5	58.6	0.145	5.286	20	49	11418	0.01	2.5
Pott's	9.9	27.4	0.083	4.676	29	64	11020	0.04	8.5
Upper Mud	11.0	66.9	0.127	6.290	34	1	11450	0.26	0.0
Cemetery	19.1	98.0	1.436	2.150	30	9	11470	0.14	2.0
Ellerbee	20.8	88.7	0.215	7.622	24	1	12167	0.09	3.5
Goose	39.4	100.0	0.200	15.169	34	11	12899	0.17	0.0

Notes:

1 Mean stream water concentrations recorded between June 2008 and July 2009.

2 Measure of cumulative heavy metal loading in July 2009 sediment samples.

3 Stream reach-averaged median sediment grain size based on surveys conducted in June 2007 and June 2009 [20], [21]. Low values indicate dominance by smaller sand particles. High values indicate dominance by larger pebbles and granules.

4 Total degree-days calculated with daily minimum and maximum temperatures using the double triangle method [56] and data taken between May 2007 and June 2007 [20].

5 Flashiness estimated from changes in hourly discharge between May and June 2007 [20].

6 Mean number of macroinvertebrate species belonging to *Ephemeroptera*, *Plecoptera*, and *Trichoptera* found in 2006 and 2007 surveys [21]. EPT richness is often used as an indicator of water quality; EPT species tend to occur in clean, well oxygenated waters [57].

### Sediment collection

We collected sediments from streams on four sediment sampling dates – sampling date 1 (June 2008), 2 (December 2008), 3 (June 2009), and 4 (July 2009). All streams were open to public access and did not require permits for collecting sediments. Mud Creek and Lower Mud were dry on sampling date 4, so sediments were not collected from those streams on that date. To collect sediments, we randomly selected five points along each ∼100 m study reach. We then demarcated a 2 m segment of streambed (i.e., entire wetted width) upstream and downstream of each selected point and used PVC corers (6.35 cm diameter) to take multiple sediment cores in each sampling area until a total volume of at least 4,024 cm^3^ was collected. Sediment cores ranged from 1 to 12 inches in vertical depth, depending on the depth of the stream bedrock. Sediment cores were sieved (2 mm opening) and composited in the field, resulting in a single composited sample from each site on each date. Sediment subsamples for molecular analyses and denitrification potentials were kept on ice for transport to the laboratory and then stored at −80°C and 4°C, respectively. Denitrification potentials were measured within 48 hours of sample collection. It is worth noting that streamwater temperatures are nearly always above 4°C in these streams, so denitrification potentials may have been negatively affected by the sudden temperature change associated with transport and storage conditions. Although not ideal in some respects, it was important to keep samples cool to minimize changes to bacterial abundances following sampling.

### Water chemistry

We collected streamwater samples from each site on the same day of sediment sampling and at least once per month from June 2008 to July 2009. Samples were field filtered through Whatman GF/F filters (Whatman, Piscataway, NJ, USA) and kept on ice for transport to the laboratory. All samples were stored at 4°C and analyzed for nitrate and total organic carbon (TOC). We measured nitrate with an ion chromatograph equipped with an AS18 anion column and KOH eluent generator (Dionex, Sunnyvale, CA, USA). We measured TOC as non-purgeable organic carbon with a TOC analyzer (TOC-V CPH, Shidmadzu Corporation, Kyoto, Japan). For all statistical analyses of nitrate and TOC concentrations, we used all available measurements from the month of each sediment sampling date to calculate a mean value for that particular site and sediment sampling date.

### Sediment heavy metals

While pharmaceuticals, herbicides, and other toxic chemicals can also contaminate urban streams, we chose to focus primarily on heavy metals as an indicator of overall contamination intensity, because previous work has documented denitrifier community shifts in response to heavy metal contamination [22] and heavy metal concentrations are relatively easy to measure. We measured the concentration of nine heavy metals, including silver (Ag), aluminum (Al), arsenic (As), cadmium (Cd), chromium (Cr), copper (Cu), nickel (Ni), lead (Pb), and zinc (Zn), in sediments collected in June 2009.

To measure heavy metal concentrations, sediments were re-sieved (1 mm opening), dried at 60°C for 48 hours, and then weighed out into three replicate 1 g subsamples per site per date for digestion (EPA method 3050B) [23]. Digestion involved adding 10 mL of 50% nitric acid, heated at 95°C, followed by another 5 mL of nitric acid (also heated to 95°C) to each sample. 10 mL of hydrogen peroxide were then added before filtering (Whatman #41). Digested samples were analyzed for trace metals by inductively coupled plasma-mass spectrometry (Perkin-Elmer Elan 6000 ICP-MS, Perkin-Elmer, Waltham, MA, USA). For every 35 samples analyzed, we also processed three replicates of certified reference materials STSD-3 (NRC, Ottawa, Canada) and two method blanks.

To correct for differences in organic carbon content among sediment samples, we standardized all heavy metal concentrations using ash-free dry mass (AFDM). AFDM is the amount of dry mass of a sediment sample that is organic and can, therefore, be combusted. To determine AFDM values, we weighed out three replicate 5 g subsamples per site per date, dried at 60°C for 48 hours, weighed to get dry mass, combusted at 400°C for 4 hours, and weighed again to get combusted mass [24]. The difference between dry and combusted mass, divided by dry mass is AFDM. Standardization is necessary for comparing concentrations of substances, like heavy metals and organic pollutants, in sediments that vary in their physio-chemical properties. While there are many normalizers available, organic carbon content is often used for standardizing heavy metal concentrations [25], [26].

In addition to the concentrations of individual heavy metals, we also calculated an additional metric (total metals) to represent the cumulative heavy metal load in sediments by categorizing each heavy metal concentration into a quintile category (i.e., 1 to 5) and then summing quintile values across all nine measured heavy metals for each site. Quintile values give equal weight to all metals, as opposed to a simple sum of concentrations, which would weigh metals with the highest concentrations (that may not be the most toxic) most heavily.

### Sediment bacterial community composition

The focus of this study was on assessing variability in community composition among sites, rather than within each site. We therefore extracted DNA from sediment subsamples taken from field composited (per site and sampling date) sediment cores. Extractions were done using PowerSoil kits (MoBio Laboratories, Carlsbad, CA, USA), according to manufacturer instructions. Samples collected on sampling dates 1 and 2 were extracted in triplicate, while those collected on sampling dates 3 and 4 were extracted in duplicate. We used extractions from sampling dates 3 and 4 to characterize overall bacterial communities and extractions from sampling dates 1, 2, 3, and 4 to characterize denitrifier communities.

We amplified bacterial 16S rRNA genes with the bacterial primer set 8F (5′-AGAGTTTGATCCTGGCTCAG, HEX labeled) [27] and 1389R (5′- ACGGGCGGTGTGTACAAG) [28] using Apex 2× Taq Master Mix (Genesee Scientific, San Diego, CA, USA). Each of 28 polymerase chain reaction (PCR) cycles consisted of 45 seconds at 94°C, 45 seconds at 58°C, and 90 seconds at 72°C. Three separate 16S rRNA PCRs were done for each extraction (i.e., two extractions per site on each date).

We amplified denitrifier DNA with two functional gene primer sets – *nirK* and *nosZ*. The *nirK* primer set was nirK1F (5′- GG(A/C)ATGGT(G/T)CC(C/G)TGGCA, FAM labeled) and nirK5R (5′-GCCTCGATCAG(A/G)TT(A/G)TGG) [29]. The *nosZ* primer set was nosZ-F (5′- CG(C/T)TGTTC(A/C)TCGACAGCCAG, FAM labeled) and nosZ1622R (5′- CGC(G/A)A(C/G)GGCAA(G/C)AAGGT(G/C)CG) [30]. We used Apex 2× Taq Master Mix (Genesee Scientific) for both *nirK* and *nosZ* denitrifier PCRs.

*nirK* is a functional gene that encodes the copper containing form of nitrite reductase, which catalyzes the first step in the denitrification pathway. Each of 33 *nirK* PCR cycles consisted of 30 seconds at 95°C, 30 seconds at 46°C, and 45 seconds at 72°C. Primers were also tested for the amplification of *nirS*, which encodes an alternate form of nitrite reductase (i.e., containing cytochrome *cd*_1_), but repeated amplification difficulties with the primer set (nirS1F and nirS6R) [29] prevented their inclusion in this study. *nosZ* is a functional gene that encodes for nitrous oxide reductase, which catalyzes the last step in the denitrification pathway. Each of 35 *nosZ* PCR cycles consisted of 30 s at 94°C, 60 s at 53°C, and 60 s at 72°C. Each DNA extraction (two to three per site by sampling date) was amplified in triplicate PCRs for both denitrifier primer sets.

Resulting PCR products were composited (i.e., three PCRs per extraction combined to yield two to three PCR product pools per site per date for each primer set), cleaned with Qiaquick PCR purification kits (Qiagen, Germantown, MD, U.S.A.), checked for appropriate sizes by agarose gel electrophoresis, and then used to generate terminal restriction fragment length polymorphism (TRFLP) profiles with either endonuclease Msp I for 16S rRNA products, Hae III for *nirK* products, or Mn lI for *nosZ* products. Endonucleases were chosen based on test runs with different endonucleases; we used endonucleases that yielded the largest number of fragments, as visualized on 1% agarose gels, for that particular PCR product type. All endonucleases were from New England Biolabs (Ipswich, MA, USA). Subsequent electrophoresis runs were done with an ABI Prism 3100 genetic analyzer (Applied Biosystems, Foster City, CA, USA) using a 1000 ROX size standard (Applied Biosystems).

To process TRFLP data, we used T-REX software [31] to determine a baseline fluorescence threshold for filtering true peaks from background noise and to align terminal restriction fragments. T-REX uses a filtering algorithm that eliminates peaks that do not meet a user-specified standard deviation limit [32]. We used one standard deviation in peak area as the limit. Each peak corresponds to a terminal restriction fragment length and represents an operational taxonomic unit (OTU). Following the filtering procedure, we aligned fragments by using a clustering threshold of 0.5 base pair [33]. Any OTUs present in less than 5 percent of samples were eliminated. For all subsequent analyses, we transformed the processed TRFLP data to presence-absence matrices and averaged replicates (i.e., two to three replicates for each site, date, and primer set combination) by the following logic - OTUs found present in at least one replicate were recorded as present (1), while those not found in any replicate were recorded as absent (0) at that site on that particular date. While this method of scoring OTU presence may have a higher risk of introducing biases associated with contamination and/or incomplete digestion (as opposed to requiring presence in at least two replicates, for instance), we chose to use this more lenient approach to scoring OTU presence to minimize the risk of throwing out ‘real’ but rare OTUs that have already survived the previous quality control step (i.e., OTUs present in <5% of samples eliminated).

The TRFLP method is a cost-effective tool that has been accepted as an appropriate and efficient means of assessing overall dissimilarity among microbial community composition [28], [34], [35].

### Denitrification potential

We used denitrification enzyme activity (DEA) assays to measure denitrification potentials [36] of sediments collected from all four sampling dates. DEA assays are short laboratory incubations conducted at room temperature under optimal conditions (i.e., anoxia and unlimited substrate availability). Five replicate incubation slurries were prepared for each stream on each sampling date by weighing 10 g subsamples of sediment into Erlenmeyer flasks (125 mL) and adding 20 mL of stock media solution with 0.72 g potassium nitrate, 0.5 g glucose, and 0.125 g chloramphenicol in 1 L of double de-ionized water.

After adding media, flasks were topped with butyl rubber stoppers (Grace, Deerfield, Illinois, U.S.A.) to achieve airtight conditions and made anaerobic by three successive cycles of evacuation and nitrogen gas (N_2_) flushing. The incubation was initiated by adding 10 mL of acetylene (C_2_H_2_) gas to each flask. C_2_H_2_ inhibits nitrous oxide (N_2_O) reduction (the last step of the denitrification pathway), allowing N_2_O to accumulate in the flask headspace. Flasks were continuously shaken (125 rpm) on reciprocal shakers during the 90 minute incubation. Gas samples were taken at the start of the incubation and every 30 minutes thereafter. Chloramphenicol inhibits the synthesis of new enzymes and bacterial communities are unlikely to change significantly during the short incubation period [37].

DEA assays provide estimates of maximum denitrification rates achievable by the extant community, given optimal conditions, but without allowing sufficient time for shifts in denitrifier community structure due to growth or the synthesis of new denitrification enzymes. The rates measured by DEA assays are, therefore, a function of two key aspects of each sample: 1) the concentration of denitrification enzymes, which reflects stream conditions at the time of sampling, and 2) the structure of the denitrifier community, which reflects a legacy of stream conditions over a period of time leading up to the time of sampling. DEA assays are widely used as a valid means of comparing denitrification rates among sites [38].

We measured N_2_O concentrations using a Teledyne Tekmar 7000 headspace autosampler (Teledyne Tekmar, Mason, Ohio, U.S.A.) to inject samples into a Shimadzu GC-17A ver.3 gas chromatograph with a Porapak Q column and electron capture detector (injector temperature = 380°C, column temperature = 80°C, detector temperature = 340°C, with N_2_ carrier gas). We used Bunsen coefficients to determine N_2_O concentrations in each sample and calculated rates of N_2_O production as the average rate observed over any 30 minute interval. N_2_O production rates were then divided by the dry mass of sediments in the flask to calculate denitrification potentials (ng N/g sediment/hr). Statistical analyses of denitrification potentials were based on averaged potentials across all five DEA assay replicates done for each site on each sampling date.

### Data analyses

To explore relationships among measured variables, we used R 2.11.1 (R Core Development Team) software to conduct simple linear regressions. To equalize variances and normalize residuals, denitrification potential, nitrate, TOC, and heavy metal values were natural log transformed, while watershed % IC and % development were arcsine square root (arcsq) transformed prior to this and all subsequent data analyses.

To test the null hypothesis of no difference in log-denitrification potential, log-nitrate, and log-TOC among streams, we used R to conduct repeated measures analysis of variance (rmANOVA) with watershed impervious cover as a factor. That is, we categorized streams *a priori* into three groups: low (<3% IC; Mud Creek and Stony), intermediate (9 to 10% IC; Lower Mud and Pott's), and high (>10% IC; Upper Mud, Cemetery, Ellerbe, and Goose) impervious cover streams (Table 1).

To test the null hypothesis of no difference in community composition among streams, we conducted permutational multivariate analysis of variance (perMANOVA) [39] in R using the adonis function in vegan [40]. perMANOVA is similar to redundancy analysis [41] and calculates a pseudo *F*-statistic by comparing the total variance explained by sample identity (i.e., % IC groups) to that explained by random permutations of sample identities. To avoid pseudoreplication, permutations (n = 9,999) were constrained by sampling date. Calculations were based on presence-absence matrices and Jaccard distance measures.

To visualize differences in community composition among sites, we created non-metric multidimensional scaling (NMS) ordinations in R using the nmds function in ecodist [42]. We used Jaccard distance measures, random starting configurations, and 200 runs with real data for each ordination. NMS creates a mapping of samples into a reduced ordination space that preserves the rank order of ecological distances among samples. Classical hypothesis testing can be conducted on ordination scores if test assumptions are met [43], [44].

Following the ordination, we analyzed potential correlations between bacterial community composition and urbanization intensity by regressing mean ordination scores for each primer set against arcsq-transformed % IC [45].

We built a linear mixed-effects (LME) model of log-denitrification to compare the explanatory power of substrate concentration versus denitrifier community composition variables. We used LME because the method can account for non-independence of errors (e.g., those rising from pseudoreplication) by differentiating between fixed versus random effects [46]. LME analyses were done using the lme function (package nlme in R) [47] with maximum likelihood estimation. We specified log-nitrate, log-TOC, and *nirK* and *nosZ* ordination scores as fixed effects and sampling date, nested within site as a random effect. We tested auto-correlation of residuals with a simple autoregressive model of order 1 [48]. To assess model fit, we calculated a pseudo-R^2^ based on a likelihood ratio test [49].

We started with a complete model that included log-nitrate, log-TOC, all three *nirK* ordination axes scores, and all three *nosZ* ordination axes scores. Substrate concentrations were means of all available measurements from the month of each sediment sampling date. We simplified the complete model by sequentially removing the least significant term and using a likelihood ratio test to compare the deviance of the simpler versus more complex model [48]. If removal led to an insignificant change in deviance, we eliminated the term from all further evaluations. We also calculated the Aikaike information criterion (AIC) to compare models. AIC penalizes against additional parameters and decreases when more of the residual variation in log-denitrification is explained.

We built a second LME model with interaction terms to explore potential interactions between substrate concentration and denitrifier community composition. We used the same random effects structure and model simplification approach as for the first model. For the second LME model, the complete model included log-nitrate, log-TOC, and the two best performing (based on results of the first LME model) community composition parameters as single terms, along with all possible two-way interaction terms. We did not include more single terms and interactions because of the relatively small dataset size (n = 30).

## Results

### Study stream characteristics

Study streams varied widely in terms of watershed impervious cover (0.5 to 39.4%), and watershed development (4.4 to 100%) (Table 1). Monthly water sampling over the course of this research effort also revealed a wide range of nitrate concentrations (from 0.017 to 1.706, mean: 0.327 mg NO_3_-N L^−1^) and TOC (from 1.298 to 35.525, mean: 6.350 mg C L^−1^) concentrations across sites. Study streams also had a wide range of heavy metal concentrations (Table S1). Total metals, a measure of cumulative heavy metal loading, ranged from 16 to 34. Table S2 provides data on the mean water and mean organic carbon content for sediments collected from study streams.

Macroinvertebrate surveys conducted within these same study streams revealed substantial declines in the diversity of sensitive macroinvertebrate species within the families *Ephemeroptera*, *Plecoptera*, and *Trichoptera* (EPT) [21] in streams draining more highly urbanized watersheds [20] (Table 1 and Table S3). Streams with higher % IC and % development also had significantly higher total summer degree-days. Relationships between heavy metals and urbanization were significantly positive for Ni and marginally significantly positive for Al, Cd, and Pb.

### Overall bacterial and denitrifier communities

The mean relative fluorescence units for OTUs that were present in less than 5 percent of samples and therefore eliminated was 107 for overall bacterial (n = 101 OTUs), 17 for *nirK* denitrifier (n = 119 OTUs), and 20 for *nosZ* denitrifier (n = 139 OTUs) communities. For reference, the mean relative fluorescence units for OTUs retained after data processing was 371 for overall bacterial, 301 for *nirK* denitrifier, and 1054 for *nosZ* denitrifier communities. Microbial community composition was significantly affected by watershed urbanization. Across these eight streams, the composition of overall bacterial, *nirK* denitrifier, and *nosZ* denitrifier communities clustered into the three groups we defined based on % IC: low, intermediate, and high impervious cover streams (overall bacterial: F_2,11_ = 1.52, *p*-value = 0.032 ; *nirK* denitrifier: F_2,27_ = 2.46, *p*-value<0.001 ; *nosZ* denitrifier: F_2,27_ = 1.37, *p*-value<0.001).

**Figure 2:**
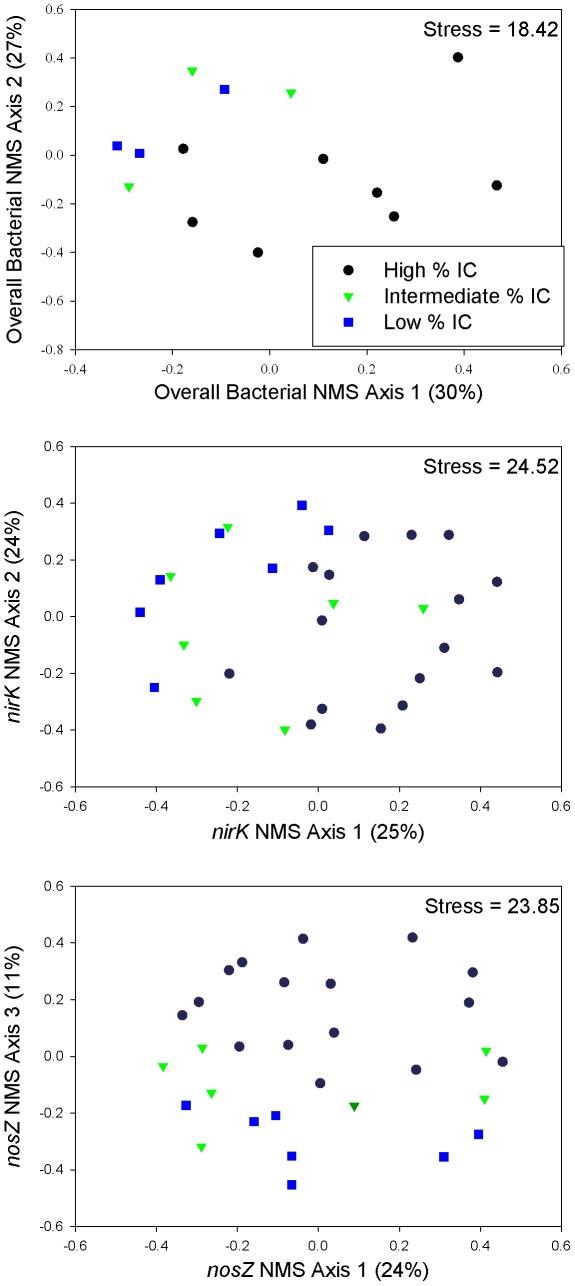
Non-metric multidimensional scaling (NMS) ordination of overall bacterial and *nirK* and *nosZ* denitrifier communities based on presence-absence TRFLP (terminal restriction fragment length polymorphism) data. Each point represents the community in a study stream on a particular sampling date. Black circles are streams in watersheds with high percent impervious cover (% IC). Green triangles are streams in watersheds with intermediate % IC. Blue squares are streams in watersheds with low % IC. Values given in parentheses following axes titles are estimated R^2^ values for individual axes. All three NMS ordinations had a final solution with three dimensions. Total R^2^ values for overall bacterial, *nirK*, and *nosZ* ordinations were 0.70, 0.55, and 0.59, respectively.

In the NMS ordinations, communities from low and intermediate impervious cover streams were separated from communities from high impervious cover streams, as indicated by the separation along axis 1 for the overall bacterial ordination, axis 1 for the *nirK* ordination, and axis 3 for the *nosZ* ordination (Fig. 2).

**Figure 3:**
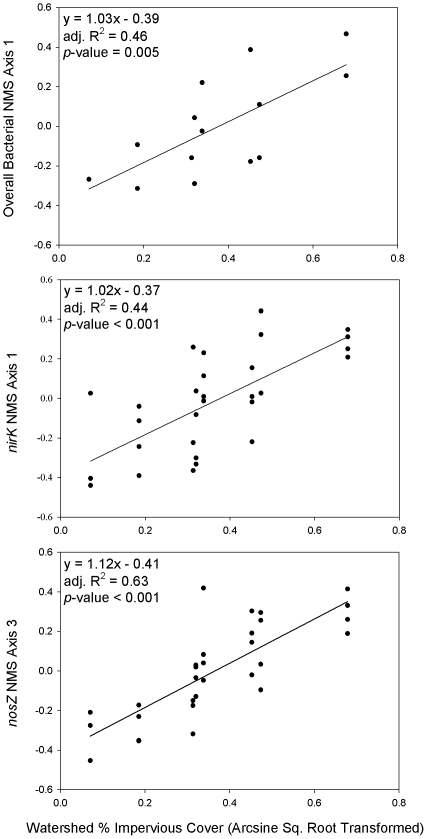
Linear regressions of NMS ordination axes scores against arcsine square root transformed percent impervious cover.

The linear regression of overall bacterial NMS axis 1 against arcsq-transformed % IC was highly significant (F_1,12_ = 11.88, R^2^ = 0.50, *p*-value = 0.005) (Fig. 3). The same was also true for the linear regression of *nirK* NMS axis 1 versus arcsq-transformed % IC (F_1,28_ = 23.77, R^2^ = 0.46, *p*-value<0.001). The linear regression of *nosZ* NMS axis 3 against arcsq-transformed % IC was also highly significant (F_1,28_ = 49.86, R^2^ = 0.64, *p*-value<0.001).

### Denitrification potentials

Denitrification potentials ranged between 41 and 561 ng N g sediment^−1^ hr^−1^, with a mean DEA of 195 ng N g sediment^−1^ hr^−1^ (Table S4). Watershed land cover was not associated with significant differences in log-denitrification potentials (rmANOVA: F_2,3_ = 1.35, *p*-value = 0.44). Log-denitrification potentials were also not significantly different across sampling dates (rmANOVA: F_3,13_ = 3.02, *p*-value = 0.07).

### Linear mixed-effects models

There was no evidence of autocorrelation of observations within groups, suggesting that errors were normally distributed within groups (i.e., sampling dates within sites). In the first LME model without interaction terms, log-nitrate, log-TOC, and all *nirK* ordination scores were removed from the final, best fitting model (Table 2, Table S5). The final model included all three *nosZ* ordination scores and captured an estimated 38% of the variation in log-denitrification. Although nosZ ordination axis 3 was not a significant term, its removal increased AIC and decreased pseudo-R^2^, so we kept the term in the final model. Compared to the intercept only model (AIC = 69.51), the final model (AIC = 61.21) had lower deviance (*p*-value = 0.003).

**Table 2:** Results from an ANOVA conducted to determine the significance of fixed effects on the final linear mixed-effects model (without interactions) of log-denitrification.

Parameter	Num DF	Den DF	*F*-value	*p*-value
Intercept	1	19	956.38	<0.001
*nosZ* ordination axis 1	1	19	7.875	0.011
*nosZ* ordination axis 2	1	19	4.636	0.044
*nosZ* ordination axis 3	1	19	3.403	0.081

Note: While nosZ ordination axis 3 was not a significant term, its removal increased AIC (see Table S5) and decreased pseudo-R^2^, so we kept the term in the final model.

In the second LME model with two-way interaction terms, the starting model included log-nitrate, log-TOC, and nosZ ordination axes 1 and 2, along with all two-way interactions. We used the ordination axes 1 and 2 because they explain a larger proportion of the variation in composition than any other combination of axes. Log-nitrate, log-TOC, and all two-way interactions, except that between log-TOC and *nosZ* ordination axis 1, were deleted from the final, best fitting model (Table 3, Table S6). The final model (AIC = 57.62) captured an estimated 45% of the variation in log-denitrification and had lower deviance than the intercept only model (AIC = 69.51) (*p*-value<0.001).

**Table 3:** Results from an ANOVA conducted to determine the significance of fixed effects on the final linear mixed-effects model (with interactions) of log-denitrification.

Parameter	Num DF	Den DF	*F*-value	*p*-value
Intercept	1	19	796.17	<0.001
*nosZ* ordination axis 1	1	19	9.420	0.006
*nosZ* ordination axis 2	1	19	5.899	0.025
*nosZ* ordination axis 1: log-TOC	1	19	6.977	0.016

## Discussion

Watershed urbanization imposes numerous stressors on stream inhabitants, including increased contaminant concentrations, stream water temperatures, and hydrologic disturbance [1]. Our results suggest that these urban stressors can drive changes in the composition of bacterial communities in streams. Given the key roles bacteria play in the biogeochemical cycling of nutrients and organic matter [4], such compositional changes could affect ecosystem functioning, particularly if the composition or activity of specific functional groups within the overall bacterial community are altered by urban inputs or stressors. In our study, denitrifier communities sorted along the urbanization gradient, regardless of whether nitrite (*nirK*) or nitrous oxide (*nosZ*) reductase primers were used to characterize communities. Knowledge of denitrifier community composition, in turn, greatly improved our ability to capture observed variations in denitrification potential.

### Watershed % IC is a good predictor of bacterial community composition in streams

Our study streams encompassed chemical (nutrients, heavy metals), physical (stream water temperatures) and hydrologic (flashiness) gradients that were generally positively associated, though not always significantly, with watershed % IC. While several factors are likely to regulate bacterial community composition, we found that a single variable, watershed % IC, was strongly and significantly correlated with overall bacterial and denitrifier community composition across our study streams. Moreover, communities from low, intermediate, and high % IC streams were significantly different from one another.

Clearly, watershed land cover itself is not directly driving differences in bacterial community composition. Rather, % IC provides an integrative measure of the intensity with which watershed urbanization may be altering numerous different measured and unmeasured aspects of stream conditions, which are, in turn, regulating bacterial community composition. Given the limited number of sites in this study, we cannot identify the specific urban impacts primarily responsible for observed patterns in bacterial community composition. We can, however, conclude that watershed % IC is a good predictor of bacterial community composition in these streams and that watershed urbanization has the potential to strongly affect bacterial community composition, albeit through as yet unidentified pathways. Further support for these conclusions can be found in the consistency with which the composition of different groups of bacteria (i.e., overall bacteria, *nirK* denitrifiers, and *nosZ* denitrifiers) appeared to sort along the urbanization gradient identified in this study.

One substantial limitation in this study arises from unmeasured covariates that may present potential confounding factors (i.e., factors other than watershed urbanization) that may play some role in driving observed differences in bacteria community structure in these study streams. Thus, further studies, using both observational and experimental approaches, are needed to definitively describe the relationships between watershed urbanization and bacterial community composition in receiving streams. Depsite this limitation, we feel this study provides intial insights into the potential for stream bacterial communities to respond to urban impacts in a similar manner as larger fish and insect communities.

### Denitrifier community composition helps explain denitrification potential

Model results indicate that denitrifier community composition may be more important than substrate supply in driving denitrification rates in our study streams. The first LME model captured an estimated 38% of the variation in denitrification potential among streams and included three composition parameters, but no substrate parameters. The second LME model captured an estimated 45% of the variation in denitrification potential among streams and included two composition parameters and an interaction term between composition and organic carbon. The interaction suggests that the capacity of communities to utilize available carbon substrates depends, in part, on which denitrifier taxa are present.

These results suggest that one important pathway through which watershed urbanization may alter stream ecosystem functioning is by changing bacterial community composition in streams. That is, community composition can directly influence rates of functioning, independent of environmental factors. It remains unclear whether the observed links between urbanization-driven shifts in denitrifier community composition and denitrification potential extend to other microbe-mediated ecosystem processes in streams, such as decomposition and carbon cycling. Future studies could address this research gap by characterizing different functional groups of bacteria and measuring their function rates in streams with varying degrees of watershed urbanization.

Study findings also provide novel insight into the potential impacts of watershed urbanization on the production of nitrous oxide gas from incomplete denitrification in streams. Nitrous oxide is a potent greenhouse gas that contributes to climate change and stratospheric ozone depletion. Although we did not measure nitrous oxide emissions, we did examine patterns of distribution for nitrous oxide reducing denitrifiers (i.e., *nosZ* denitrifiers) in relation to urbanization intensity. Our findings suggest that the composition of *nosZ* denitrifier communities may be strongly affected by urbanization, which may speculatively help explain recent observations indicating that urban streams may release more nitrous oxide to the atmosphere than non-urban streams [50].

### Conclusions

Microbes are the most ubiquitous, abundant, and diverse group of organisms on Earth [51]. Our understanding of how ecosystems respond to land-use change would be incomplete without the microbial perspective. Ecologists generally agree that shifts in plant community composition can alter ecosystem process rates [52], [53]. The same could be true for microbial community composition, yet ecosystem modeling efforts have largely ignored microbial community composition [54]. Given advances in molecular technology, we can now question the assumption that microbial communities are resistant to anthropogenic disturbances and that they can be adequately represented as ‘black boxes’ responding only to substrate supply in ecosystem models [55].

This study demonstrates that incorporating data on bacterial community composition, even relatively low resolution data from a molecular fingerprinting method, can drastically improve our ability to model ecosystem process rates, particularly under realistic scenarios of environmental degradation. To identify and rank specific drivers of bacterial community change in urban streams, we need to survey a larger number of streams and collect more comprehensive data on factors like contamination, temperatures, and hydrology. This information would improve our ability to understand the detrimental impacts of urbanization on microbial community structure and function. Future research should focus on understanding not only the causes of microbial community change in human impacted ecosystems, but also how these communities differ in resistance and function.

## Supporting Information

Table S1 Heavy metals concentrations in stream sediments.
(DOC)

Table S2 Stream sediment mean water and organic carbon content.
(DOC)

Table S3 Correlations (Pearson's *r*) between watershed metrics and measured stream characteristics across all sites and dates.
(DOC)

Table S4 Denitrification potential.
(DOC)

Table S5 Non-significant terms deleted from the complete version of the first linear mixed-effects model of log-denitrification (without interaction terms).
(DOC)

Table S6 Non-significant terms deleted from the complete version of the first linear mixed-effects model of log-denitrification (with interaction terms).
(DOC)
