# Supplementary material for: Watershed Urbanization Alters the Composition and Function of Stream Bacterial Communities
Source: PLoS One. 2011 Aug 12;6(8):e22972. doi: 10.1371/journal.pone.0022972 (PMC3155513; doi:10.1371/journal.pone.0022972)
Supplement: Table S1 — Heavy metals concentrations in stream sediments. (DOC) [file pone.0022972.s001.doc]

| Stream | Ag (μg/g) | Al (mg/g) | As (μg/g) | Cd (μg/g) | Cr (μg/g) | Cu (μg/g) | Ni (μg/g) | Pb (μg/g) | Zn (μg/g) |
| --- | --- | --- | --- | --- | --- | --- | --- | --- | --- |
| Mud Creek | 0.148 | 29.51 | 0.646 | 0.022 | 3.410 | 6.057 | 1.978 | 3.866 | 18.25 |
| Stony | 0.013 | 29.19 | 1.781 | 0.043 | 8.367 | 108.6 | 1.391 | 7.629 | 67.26 |
| Lower Mud | 0.017 | 24.81 | 1.492 | 0.029 | 12.01 | 7.152 | 3.745 | 4.855 | 20.18 |
| Pott’s | 0.029 | 45.83 | 0.417 | 0.025 | 17.41 | 90.38 | 2.611 | 10.25 | 37.35 |
| Upper Mud | 0.297 | 44.00 | 1.660 | 0.030 | 14.75 | 9.278 | 5.117 | 8.064 | 23.76 |
| Cemetery | 0.025 | 48.73 | 0.263 | 0.040 | 6.049 | 55.18 | 3.406 | 28.07 | 44.61 |
| Ellerbee | 0.146 | 35.83 | 0.470 | 0.028 | 12.37 | 5.652 | 5.477 | 5.740 | 14.00 |
| Goose | 0.041 | 50.90 | 0.858 | 0.054 | 8.218 | 11.57 | 7.913 | 13.89 | 36.06 |
