# Supplementary material for: Watershed Urbanization Alters the Composition and Function of Stream Bacterial Communities
Source: PLoS One. 2011 Aug 12;6(8):e22972. doi: 10.1371/journal.pone.0022972 (PMC3155513; doi:10.1371/journal.pone.0022972)
Supplement: Table S2 — Stream sediment mean water and organic carbon content. (DOC) [file pone.0022972.s002.doc]

| Stream | Water content (%) | | Organic carbon content (%) | |
| --- | --- | --- | --- | --- |
|  | Mean | Standard deviation | Mean | Standard deviation |
| Mud Creek | 18.70 | 0.22 | 0.88 | 0.11 |
| Stony | 25.77 | 13.37 | 2.24 | 0.65 |
| Lower Mud | 14.88 | 0.16 | 1.15 | 0.41 |
| Pott’s | 14.90 | 3.99 | 0.69 | 0.04 |
| Upper Mud | 16.54 | 2.63 | 1.17 | 0.23 |
| Cemetery | 22.08 | 7.95 | 0.65 | 0.16 |
| Ellerbee | 18.50 | 4.80 | 0.46 | 0.31 |
| Goose | 16.02 | 2.31 | 0.98 | 0.21 |
