# Supplementary material for: Watershed Urbanization Alters the Composition and Function of Stream Bacterial Communities
Source: PLoS One. 2011 Aug 12;6(8):e22972. doi: 10.1371/journal.pone.0022972 (PMC3155513; doi:10.1371/journal.pone.0022972)
Supplement: Table S3 — Correlations (Pearson's r) between watershed metrics and measured stream characteristics across all sites and dates. (DOC) [file pone.0022972.s003.doc]

|  | Watershed impervious cover | Watershed  development |
| --- | --- | --- |
| Watershed impervious cover |  |  |
| Watershed development | **0.940** ^<0.001^ |  |
| Nitrate | 0.370 | 0.609 |
| TOC | 0.509 | 0.303 |
| Ag | -0.035 | -0.014 |
| Al | **0.669** ^0.070^ | 0.600 |
| As | -0.260 | -0.261 |
| Cd | 0.617 | **0.644** ^0.085^ |
| Cr | 0.311 | 0.179 |
| Cu | -0.106 | -0.144 |
| Ni | **0.856** ^0.007^ | **0.817** ^0.013^ |
| Pb | 0.594 | **0.647** ^0.083^ |
| Zn | 0.036 | 0.024 |
| Total metals | 0.605 | 0.557 |
| Degree-days | **0.894** ^0.003^ | **0.825** ^0.012^ |
| Flashiness | 0.526 | 0.588 |
| EPT richness | **-0.796** ^0.018^ | **-0.870** ^0.005^ |
| Notes: Watershed metrics were arcsine square root transformed and nitrate, TOC, and heavy metal concentrations were natural log transformed prior to analysis. Values in bold indicate correlations with *p*-values < 0.1 (given in superscript). | | |
