# Supplementary material for: Watershed Urbanization Alters the Composition and Function of Stream Bacterial Communities
Source: PLoS One. 2011 Aug 12;6(8):e22972. doi: 10.1371/journal.pone.0022972 (PMC3155513; doi:10.1371/journal.pone.0022972)
Supplement: Table S4 — Denitrification potential. (DOC) [file pone.0022972.s004.doc]

| Stream | Denitrification potential  (ng N g^-1^ hr^-1^) | |
| --- | --- | --- |
|  | Mean | Standard deviation |
| Mud Creek | 107 | 96 |
| Stony | 206 | 70 |
| Lower Mud | 76 | 16 |
| Pott’s | 128 | 75 |
| Upper Mud | 191 | 78 |
| Cemetery | 441 | 217 |
| Ellerbee | 98 | 20 |
| Goose | 262 | 201 |
