# Supplementary material for: Watershed Urbanization Alters the Composition and Function of Stream Bacterial Communities
Source: PLoS One. 2011 Aug 12;6(8):e22972. doi: 10.1371/journal.pone.0022972 (PMC3155513; doi:10.1371/journal.pone.0022972)
Supplement: Table S5 — Non-significant terms deleted from the complete version of the first linear mixed-effects model of log-denitrification (without interaction terms). (DOC) [file pone.0022972.s005.doc]

| Model | Parameters | df | AIC | Log-likelihood | Likelihood ratio | *p*-value |
| --- | --- | --- | --- | --- | --- | --- |
| 1 | log-nitrate, log-TOC, nir1, nir2, nir3, nos1, nos2, nos3 | 12 | 69.27 | -22.64 |  |  |
| 2 | log-nitrate deleted | 11 | 68.20 | -23.10 | 0.92 | 0.336 |
| 3 | nir1 deleted | 10 | 66.44 | -23.22 | 0.24 | 0.626 |
| 4 | nir3 deleted | 9 | 64.79 | -23.40 | 0.35 | 0.552 |
| 5 | nir2 deleted | 8 | 63.06 | -23.53 | 0.27 | 0.601 |
| 6 | log-TOC deleted | 7 | 61.21 | -23.60 | 0.14 | 0.705 |
| 7 | nos3 deleted | 6 | 62.69 | -25.34 | 3.48 | 0.062 |
| Notes: The parameters nir1, nir2, and nir3 refer to scores for *nirK* ordination axes 1, 2, 3, respectively. The parameters nos1, nos2, and nos3 refer to scores for *nosZ* ordination axes 1, 2, 3, respectively. Likelihood ratios and *p*-values refer to the change in deviance that resulted from the deletion of each term from the more complex model in the row above. | | | | | | |
