# Supplementary material for: Watershed Urbanization Alters the Composition and Function of Stream Bacterial Communities
Source: PLoS One. 2011 Aug 12;6(8):e22972. doi: 10.1371/journal.pone.0022972 (PMC3155513; doi:10.1371/journal.pone.0022972)
Supplement: Table S6 — Non-significant terms deleted from the complete version of the first linear mixed-effects model of log-denitrification (with interaction terms). (DOC) [file pone.0022972.s006.doc]

| Model | Parameters | df | AIC | Log-likelihood | Likelihood ratio | *p*-value |
| --- | --- | --- | --- | --- | --- | --- |
| 1 | log-nitrate, log-TOC, nos1, nos2, and all two-way interactions | 14 | 68.98 | -20.49 |  |  |
| 2 | log-nitrate : nos2 deleted | 13 | 66.99 | -20.50 | 0.01 | 0.912 |
| 3 | nos1 : nos2 deleted | 12 | 65.04 | -20.52 | 0.04 | 0.837 |
| 4 | log-nitrate : nos1 deleted | 11 | 63.05 | -20.52 | 0.01 | 0.908 |
| 5 | log-nitrate : nos2 deleted | 10 | 61.29 | -20.65 | 0.24 | 0.623 |
| 6 | log-nitrate deleted | 9 | 61.17 | -21.58 | 1.88 | 0.170 |
| 7 | log-TOC deleted | 8 | 59.51 | -21.76 | 0.34 | 0.559 |
| 8 | log-TOC : log-nitrate deleted | 7 | 57.62 | -21.81 | 0.11 | 0.741 |
| 9 | nos2 | 6 | 62.65 | -25.33 | 7.03 | 0.008 |
| Notes: The parameters nir1, nir2, and nir3 refer to scores for *nirK* ordination axes 1, 2, 3, respectively. The parameters nos1, nos2, and nos3 refer to scores for *nosZ* ordination axes 1, 2, 3, respectively. Likelihood ratios and *p*-values refer to the change in deviance that resulted from the deletion of each term from the more complex model in the row above. | | | | | | |
